# Correlation between nitrogen fixation rate and alginate productivity of an indigenous *Azotobacter vinelandii* from Iran

**Published:** 2012-09

**Authors:** R Nosrati, P Owlia, H Saderi, M Olamaee, I Rasooli, Tehrani A Akhavian

**Affiliations:** 1 Molecular Microbiology Research Center (MMRC), Shahed University, Tehran, I.R. Iran; 2 Department of Microbiology, Faculty of Medicine, Shahed University, Tehran, I.R. Iran; 3 Department of Soil Science, Faculty of Water and Soil, Gorgan University, Gorgan, I.R. Iran; 4 Department of Biology, Faculty of Science, Shahed University, Tehran, I.R. Iran; 5 Department of Plant Biotechnology, National Institute of Genetic Engineering and Biotechnology (NIGEB), Tehran, I.R. Iran

**Keywords:** Alginate, *Azotobacter vinelandii*, Nitrogenase, Nitrogen fixation

## Abstract

**Background and Objectives:**

*Azotobacter vinelandii*, a gamma-proteobacterium, is an obligate aerobic free-living gram-negative soil bacterium capable of fixing nitrogen. Oxygen transfer rate into the cell is reduced by the increase of alginate concentrations during the course of *A. vinelandii* cultivation. This phenomenon provides a low intracellular oxygen concentration needed for nitrogenase activity. The aim of this study was to design a simple strategy to explain the alginate production, cell growth and nitrogenase activity correlation in *A. vinelandii* under aerobic conditions.

**Material and Methods:**

Thirty-five different soil samples were taken from the rhizosphere of agricultural crops of Iran. Enrichment and isolation strategies were employed for microbial isolation. Physiological and biochemical characteristics were determined. Molecular identification was performed using selective *nifH-g1* primers. Alginate production and nitrogenase activity assay by each isolate of *Azotobacter* were carried out. Bacterial growth, alginate production and Nitrogenase activity were conducted by time-coursed quantitative measurements.

**Results:**

Total of 26 isolates were selected after enrichment, isolation, and screening. The isolate was identified by molecular tests as *A. vinelandii*. The highest alginate productions of 1.02 g/l and 0.91g/l were noted after 4 days in 8 isolates, cell biomass of which were estimated 4.88-5.26 g/l. Six of 8 isolates were able to fix atmospheric N_2_ on nitrogen-free medium. Rates obtained in isolates were in the range of 12.1 to 326.4 nmol C_2_H_4_ h^-1^ vial^-1^.

**Conclusions:**

Nitrogen fixation and alginate production yielded significant and positive Pearson's correlation coefficient of R^2^ = 0.760, p ∼ 0.02. Finally association between bacterial growth, alginate production and nitrogenase activity almost noticeable yielded significant and positive Pearson's correlation coefficient R2= 0.723, p ∼ 0.04.

## INTRODUCTION

*Azotobacter vinelandii* is a gamma-proteobacterium belonging to the family *Pseudomonadaceae*. It is an obligate aerobic free-living gram-negative soil bacterium capable of fixing nitrogen directly from the atmosphere that helps the plants for better grain production (1). The nitrogenase enzyme complex that catalyzes dinitrogen reduction to ammonium is composed of two highly conserved proteins: the iron (Fe) protein (encoded by the nifH gene) and the molybdenum iron (MoFe) protein (encoded by the nifDK genes) (2). Evolutionarily conserved amino acid sequences within the *nifH* gene have been oppressed to design PCR primers to detect the genetic potential for nitrogen fixation in the environment (3, 4).

Nitrogenase is highly sensitive to oxygen (2, 5), However, Nitrogen fixation occurs in *A. vinelandii* using three distinct nitrogenase systems under fully aerobic conditions that typically inactivate the nitrogenase enzyme (6). Obligate aerobes such as *A. vinelandii* are known to use two mechanisms for protecting the nitrogenase system against oxygen damage: (i) high respiration rate that the uncommonly high activities of cellular oxygen utilization, prevent the diffusion of oxygen into the cells and consequently to the nitrogenase (7), (ii) conformational protection of the enzyme or the switch-off of nitrogenase activity by shethna or FeSII protein (8). Recently, alginate formation is considered as a new protection mechanism for nitrogenase against oxygen (5).

Alginates are a family linear copolymers composed of variable amounts of (1–4) -β-D-mannuronic acid and its epimer, α-L-guluronic acid (9–11). Some bacteria especially *Pseudomonas aeruginosa* and *A. vinelandii* can produce exopolysaccharide alginate (9, 12). Alginate is important in various biotechnological and biomedical applications, e.g. for immobilizing cells in the pharmaceutical or as a stabilizing, thickening and gelling agent in food production (5). The species *A. vinelandii* seems to be the best candidate for the industrial production of alginate (13).

*Azotobacter vinelandii* produces the intracellular polymer polyhydroxybutyrate (PHB) and excretes alginate into the medium during vegetative growth. Synthesis and production of alginate and PHB by *A. vinelandii* is essential for cyst formation and differentiation. The mutant varieties of bacteria do not produce alginate and are unable to form mature cysts. The cyst is formed under unfavorable environmental conditions. The mature cysts are surrounded by two capsule-like layers containing a high proportion of the alginate. The intine (inner coat) and exine (outer coat) layers of the cyst contain different types of alginate. Under favourable conditions, the alginate coating swells and the cyst germinates (11).

The alginate extracellular accumulation acts as a barrier to oxygen diffusion or heavy metals (10). In *A. vinelandii*, the increase of alginate concentrations during the course of cultivation in culture broth can reduce the oxygen transfer rate into the cell and consequently provide a low intracellular oxygen concentration that is essential for nitrogenase activity (6, 8).

The present study was aimed to design a simple strategy to explain the correlation between alginate production, cell growth and nitrogenase activity in *A. vinelandii* under aerobic conditions.

## MATERIALS AND METHODS

### Bacterial isolation and identification

Thirty five different soil samples from the rhizosphere of agricultural crops of Iran (Tehran, Qazvin and Guilan) were transferred to laboratory. Strategies used for isolation were:

- Enrichment: For enrichment of *A. vinelandii* strains and the growth inhibition of other *Azotobacter* species, 1 g soil samples were added into 100 ml Erlenmeyer flasks containing 20 ml of Azotobacter broth medium with the following composition; K_2_HPO_4_ 0.8 g, KH_2_PO_4_ 0.2 g, MgSO_4_·7H_2_O 0.5 g, FeSO_4_·6H_2_O 0.10 g (or 0.05 g), CaCl_2_·2H_2_O 0.05 g or CaCO_3_ 20.0 g, NaMoO_4_·2H_2_O 0.05 g per liter (Adjust to pH 7.4–7.6) (14). Ethylene glycol (1%) as sole source of carbon, 0.1% phenol and cycloheximide (100 µg/ml) were added into medium and were incubated at 37°C for 2-5 days (15).
- Isolation: Serial dilutions were prepared from enrichment culture followed by streaking and incubation at 37°C. All the isolates were subcultured on selective nitrogen-free specific medium Azotobacter Agar plates and were purified.

Physiological and biochemical characteristics was performed according to Bergey's Manual of Systematic Bacteriology instructions (1), including colony morphology, the gram, cyst and PHB granules staining as well as production of pigment.

Molecular identification was performed with PCR using selective *nifH-g1* primer from *Azotobacter* (GenBank accession nos. M11579, M20568) (16):

fD1 (5'GGTTGTGACCCGAAAGCTGA-3’), rP1 (5’-GCGTACATGGCCATCATCTC-3’)

Reference strain *Azotobacter sp*. PTCC 1658 used as the control for comparison.

### Alginate production

The medium for alginate production by *A. vinelandii* contained 20 g sucrose, 0.6 g (NH_4_)_2_SO_4_, 2 g Na_2_HPO_4_, 0.3 g MgSO_4_·7H_2_O, and 6 g yeast extract per liter of distilled water at pH 7.2 (17). Erlenmeyer flasks containing 25 ml alginate production medium were inoculated with ∼10^4^CFU/ml of each isolates of *Azotobacter* and were incubated at 28°C at 180 rpm for 96 h.

### Separation of cell biomass

Separation of *A. vinelandii* cells from the culture broth was achieved in the following manner.

1. Five ml for each sample was centrifuged at 8400 rpm at 15°C for 30 min in pre weighed tubes,
2. The supernatant was removed and the residue was suspended in NaCl (5M) and Na_4_EDTA (0.5M).
3. Centrifuging as in step 1.
4. The harvested biomass was washed with deionized water and then dried at 60°C for 24 h in an oven to estimate the biomass concentration (18).

### Alginate determination

Alginate was measured by Gravimetric Method (17) as the following procedure:

1. The supernatant of previous step was removed and equal volume of ice-cold 95% ethanol was added, stirring slowly.
2. The mixture was centrifuged at 12,000 rpm at 4°C for 20 min.
3. The supernatant was carefully removed. The tubes were dried at 105°C for 24 h and alginate dry weight and concentration were determined.

### Nitrogen fixation and nitrogenase activity

The nitrogenase activity assay was carried out according to the method of acetylene reduction (19). Five ml of the Azotobacter broth medium in 12 ml vials was inoculated with ∼10^4^CFU/ml of each isolate and incubated for 48-96 h at 28 °C. Once visible growth was observed, each vial was sealed with rubber stopper. By means of a disposable plastic syringe, 10% of air from the head space (7 ml) was removed and an equal amount of acetylene was injected into vials (20, 21). Gas samples (0.7 ml) were removed after 24 h incubation, and were assayed for ethylene production with a gas chromatograph in triplicate (GOW MAC - GM 816 model). The chromatograph was fitted with Poropak N column and a H2-FID detector.

The rate of nitrogen fixation was calculated by Ravikumar formula (21) and Values were obtained nmoles C_2_H_4_ h^-1^ vial^-1^ (22):

### Bacterial growth, alginate production and Nitrogenase activity

Time-coursed quantitative measurements were carried out in Erlenmeyer flasks containing 25 ml of broth medium. 100µl of bacterial suspension were inoculated into medium and incubated at 28°C and 180 rpm. Final population of bacterial suspension was *∼*10^4^ CFU/ml of isolates with the highest alginate production (A3 and A21). The uninoculated medium used as control in each case. Sampling was carried out within 120 h. For estimation of the growth rate, 100 μl of medium were removed every 6 hours, serial dilution were prepared and were contained colonies on solid medium and the growth curve was drawn, while the measurement of simultaneous alginate production and nitrogenase activity were done at 48 h, 72 h, 96 h and 120 h in triplicates. Cell numbers at log phase of *Azotobacter* were adjusted to 10^7^ CFU/ ml.

### Statistical analysis

Data analysis and graph drawing was carried out using statistics software GraphPad Prism (v5.0.4) and SPSS (v18). We used the Bivariate Pearson's correlation to estimate the correlation between the alginate production and nitrogenase activity. Pearson's correlation measure how variables or rank orders are related, according to correlation coefficient and significance level.

## RESULTS

### Isolation and Identification

**Fig. 1:**
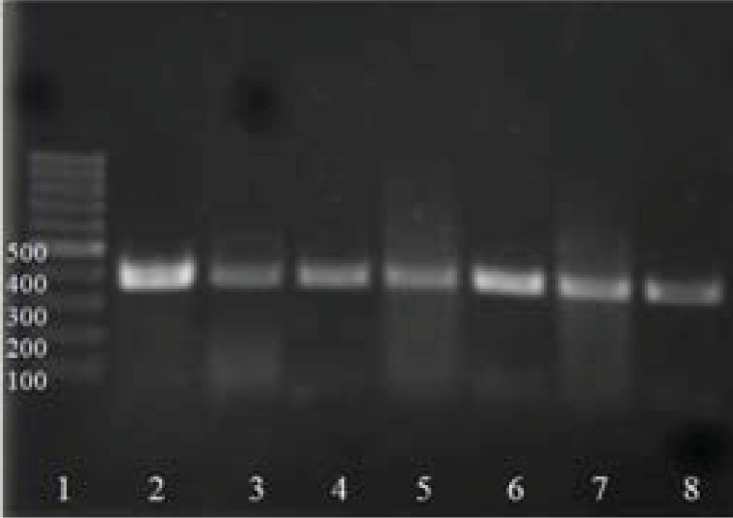
The PCR product of reference *Azotobacter* strain and *Azotobacter* isolates as follows Lanes 2 and 8, line 1 = 100 bp marker, line 2 = Reference strain Azotobacter sp. PTCC 1658, Lane 3 to 8 = Respectively; A2, A3, A5, A8, A15, A21 isolates.

Total of 26 isolates named as A1 to A26, were selected after enrichment, isolation, and screening from 35 soil samples. The *Azotobacter* isolates were identified by molecular tests with PCR by specific primers for *nifH-g1*. The PCR reaction was carried out for all the 26 isolates in order to check the presence of *nifH* gene. PCR products were about 400 bp. The results showed that there was no significant difference in banding pattern compared to the reference *Azotobacter* strains (Fig. 1). The identification of isolates was completed according to biochemical characteristics from Bergey's Manual of Determinative Bacteriology (1). The isolate was identified as *A. vinelandii*.

### Alginate production

**Fig. 2:**
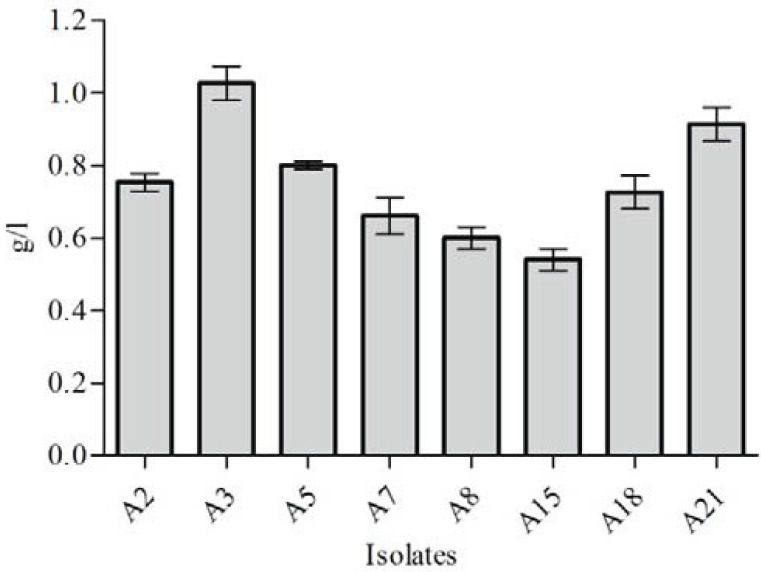
Production of bacterial alginate by selected *A. vinelandii* isolates (A2, A3, A5, A7, A8, A15, A18 and A21). The isolates were cultivated at 28°C and 180 rpm for 4 days. Values are means of triplicates.

**Fig. 3:**
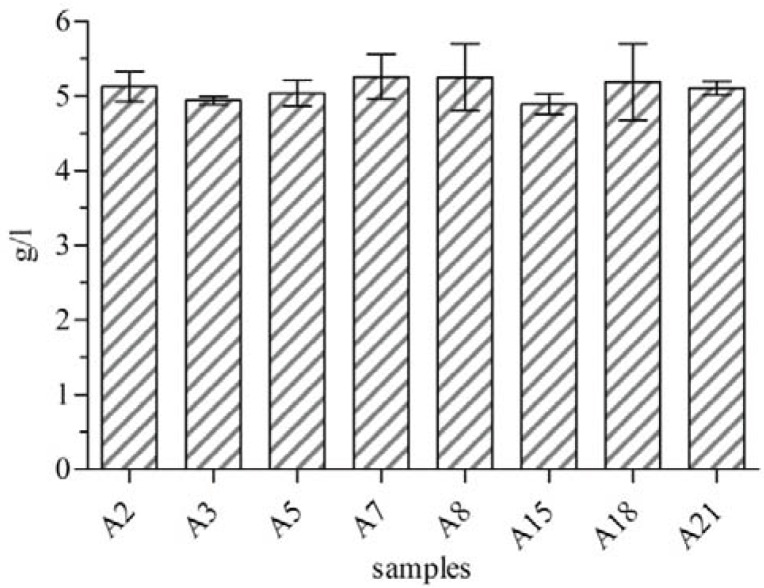
Cell biomass of selected *A. vinelandii* isolates (A2, A3, A5, A7, A8, A15, A18 and A21). Cell biomasses estimated were between 4.88 g/l and 5.26 g/l.

Among 26 isolated *A. vinelandii*, alginate production was measured in 8 isolates (A2, A3, A5, A7, A8, A15, A18, and A21) showing higher slimy and mucoid phenotype on solid medium compared to the other isolates. The highest alginate production was observed for A3 (1.02 g/l) followed by A21 (0.91 g/l) at during 4 days (Fig. 2). In this series of experiments, cell biomass of all the 8 isolates estimated were between 4.88 g/l and 5.26 g/l. The lowest rate was of A15 and both A7 and A8 had the highest values (Fig. 3).

### Nitrogen fixation and Nitrogenase activity

**Fig. 4:**
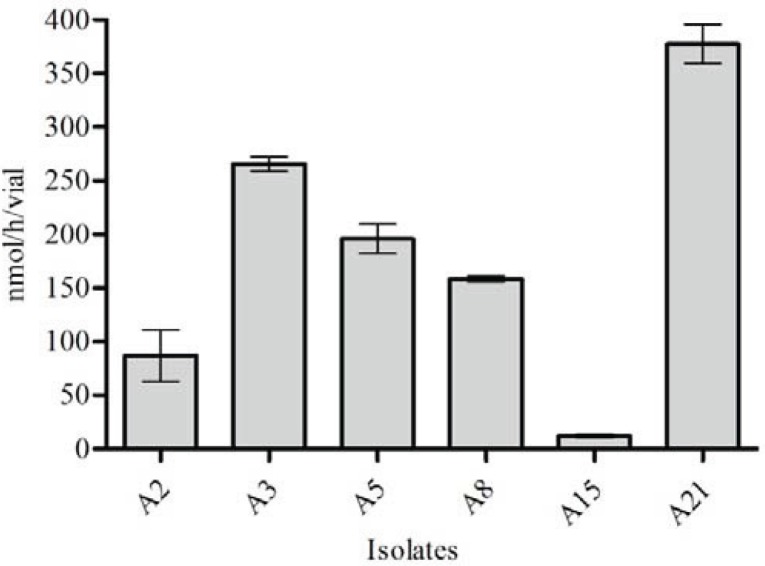
Nitrogenase activity (Acetylene Reduction Assay) by isolates that were able to fix N2 (A2, A3, A5, A8, A15 and A21). Values are means of triplicates followed by the standard deviation.

As shown in Fig. 4, among 8 isolates, only six isolates were found to be able to fix mesurable amount of atmospheric N_2_. Amounts of acetylene reduced by *A. vinelandii* isolates in samples were quite different. Rates obtained in isolates were in the range of 12.1 to 326.4 nmol C_2_H_4_ h^-1^ vial^-1^. Nitrogen fixation and alginate production yielded significant and positive Pearson's correlation coefficient (R^2^= 0.760, p ∼ 0.02).

### Bacterial growth, alginate production and Nitrogenase activity

**Fig. 5:**
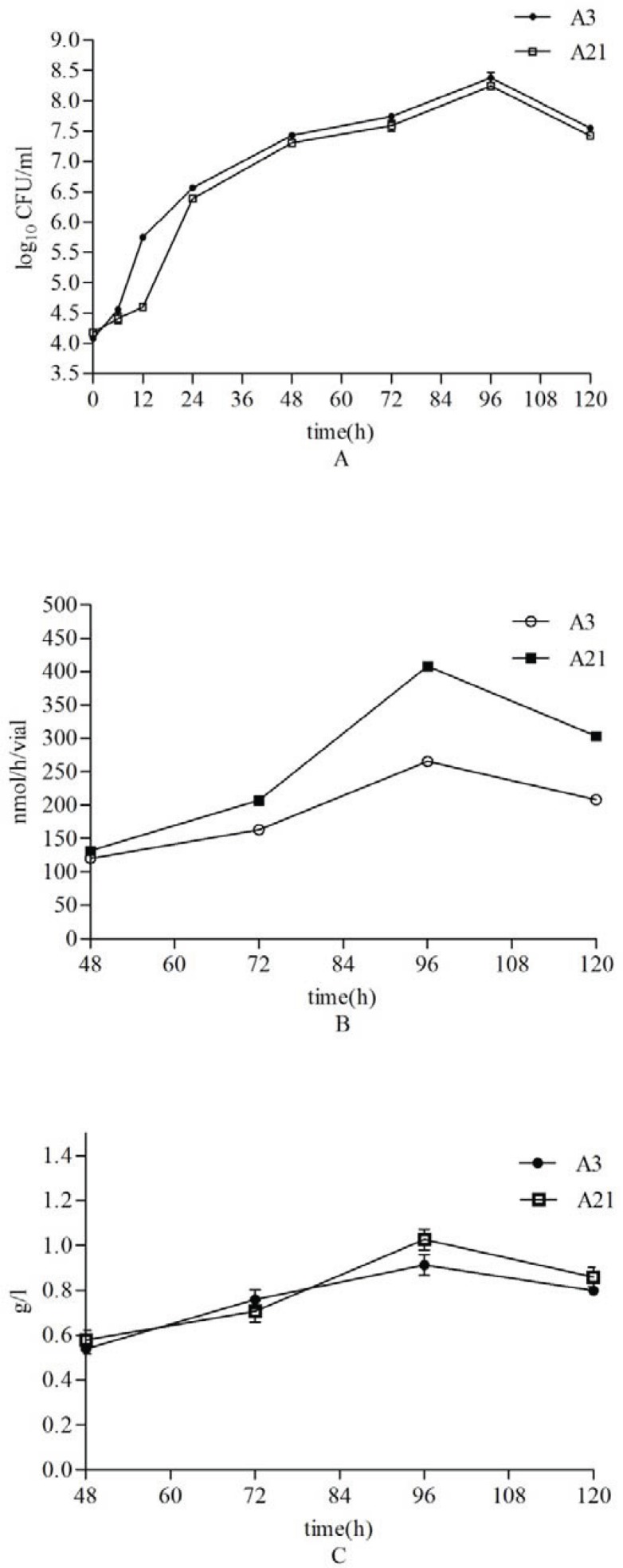
The growth curve (Panel A), nitrogen fixation (Panel B) and alginate production (Panel C) by A3 and A21 isolates. Sampling was carried out within 120 h. After 96 h, both strains arrived at stationary phase as well as the maximum levels of alginate production and nitrogen fixation. All data points are the means of three replicates. Standard errors are shown by vertical bars.

Two *A. vinelandii* isolates viz, A3 and A21, with the highest alginate production were chosen for detailed investigation to examine association between bacterial growths (which is the logarithm of CFU/ml of culture) (23), alginate production and nitrogenase activity. In both strains, almost noticeable association yielded significant and positive Pearson's correlation coefficient (R^2^= 0.723, p ∼ 0.04). The maximum levels of alginate production and nitrogen fixation for both A3 and A21 were reached after 96 h of incubation at 28°C while exponential growth phase of both strains delayed for 6 h and arrived at stationary phase after 96 h (Fig. 5A).

Alginate production and nitrogenase activity of A21 (1 g/l) were increased in comparison with A3 after 96 h (0.91 g/l) despite alginate production value of the isolate A3 was obtained higher than A21 in early experiment (section 3.2). This phenomenon provides that the increase in nitrogen-fixation dependent on alginate production and not bacterial count because the number of A21 was less than A3.

## DISCUSSION

The method employed in the present work was described by different authors as feasible for *A. vinelandii* strains isolation from other *Azotobacter* species and other free-living nitrogen-fixing soil bacteria. Claus and Hempel (24) observed that ethylene glycol in 0.1 or 0.2% (wt/vol) concentration is a very selective carbon source for *A. vinelandii*. More common *Azotobacter* species apparently cannot utilize it. It has been shown that the use of 0.1% phenol in enrichment cultures will inhibit the growth of other *Azotobacter* species and incubation at 37°C will particularly suppress the development of genus *Azomonas* and makes *A. vinelandii* selectively dominant (25). The precise identification was achieved based on universal PCR detection of the *nifH* marker gene that has been applied to describe diazotroph populations in the environment, but they could not successfully separate *Azetobacters* from other diazotrophs. Helmut *et al.* (16) confirmed the *nifH-g1* primer set that was designed to amplify *nifH* genes of *Azotobacter* species. Rajeswari and Mangai (4) reported that *nif*H-g1 primer targets *Azotobacter* spp. Combination of morphological, biochemical and molecular methods in this study confirm our identification of *Azotobacter*.

Clementi *et al.* (17) assessed the minimum alginate concentration 0.1–0.5 g/ml and speculated that gravimetric method lacks sensitivity because of precipitation of salts and peptones. In a similar study, Sabra *et al.* (6) reported 0.2–0.9 g/l for alginate production at different agitation speeds (300 to 1,000 rpm) by gravimetrical method. We obtained alginate production between 0.54–1.02 g/l by same method.

Alginate production at high concentrations by *A. vinelandii* depends on cultural conditions (26). The use of appropriate carbon and nitrogen sources, air-flow rate (agitation speed), temperature, pH allowed obtaining a maximum production. Emtiazi *et al.* (18) isolated strain AC2 with maximal production (7.5 mg/ml) in an optimized medium, 30°C and 200 rpm shaking during 4 days in 1% sucrose. Vermani *et al.* (27) suggested that *A. vinelandii* MTCC 2459 produce optimum alginate at 30°C, 110 rpm shaking, 50 g/l sucrose and 0.1 g/l NH_4_Cl at pH:7 during 72 h. Chen *et al.* (3) was obtained the largest amount of bacterial alginate at 34°C and 170 rpm shaking speed and 2% sucrose in about 110 h on optimum medium. In this work it was shown that A3 isolate produced maximum of 1.02 g/l exopolymer in media with 2% sucrose as the carbon source, at 28°C, 180 rpm shaking during 4 days.

Of several methods available for measuring nitrogen fixation, the Acetylene Reduction Assay (ARA) is simpler and faster than the other methods (28). The results presented in this paper are in agreement with the Rodelas *et al.* (29) who reported 9.70 to 257.73 nmol C_2_H_4_ h^-1^ vial^-1^ for ARA rate of *A. vinelandii*.

*A. vinelandii* has three distinct nitrogenases (3): the Mo, V and Fe-containing nitrogenase called nitrogenase 1, nitrogenase 2, and nitrogenase 3, respectively (30). The absence of vanadium in culture or mutation in *nif* gene was probably effective factors due to which we couldn't assay nitrogen fixation by isolates A7 and A18.

A possible link between alginate formation and protection of nitrogenase in this organism has not so far been examined. In fact, the biological function of alginate formation in bacteria is not fully understood (6). Nitrogen fixation is inhibited by oxygen since dinitrogenase reductase is rapidly and irreversibly inactivated by O_2_. For example at 4% oxygen level, *A. vinelandii* fixed 23.5 mg nitrogen per g sucrose supplied; at 20% oxygen the fixation was 8.1 mg nitrogen (28). *A. vinelandii* is known to produce alginate under aerobic conditions (6) while a few oxygen concentration is necessary for nitrogen fixation. Increasing biomass and alginate concentrations increase the nitrogenase activity because it reduces oxygen transferring into the cell. Sabra *et al* used transmission electron microscopy and clearly showed that the *A. vinelandii* cells grown diazotrophically at pO2 values of 20% formed capsules significant the thickness than pO2 values of 2.5% (6). The alginate concentrations and nitrogenase activity increased upon A3 and A21 reaching exponential phase. The largest values of nitrogen fixation and alginate were obtained in the termination of logarithmic phase. When both strains arrived at stationary phase, bacterial growth and nitrogen fixation values were reduced as expected.

Decreasing alginate concentration after 120 h (Fig. 5C) shows dependence of alginate on bacterial metabolism. Synthesis of alginate depends on carbon sources and it is important for cyst formation (5, 9, 11). Alginate produces during the exponential phases of growth while the cyst has form after exponential growth (6, 9). Therefore under the experimental conditions it is probable that carbon source utilized for alginate production in during log phase and respectively, alginate is used in cyst formation in stationary phase. Increased alginate elevates the viscosity of the culture broth and hence reduces aerobic conditions, leading to a decreased cell growth.

In conclusion, due to correlation between bacterial growth, alginate production and nitrogenase activity almost noticeable yielded significant, the results presented in this work hint to possible role of alginate in nitrogenase activity and it is suggested that how the quantity and quality of alginate at different O_2_ concentration regulate nitrogen fixation at physiological and genetic levels.
